# Selection and evaluation of reference genes for qRT-PCR analysis in *Euscaphis konishii* Hayata based on transcriptome data

**DOI:** 10.1186/s13007-018-0311-x

**Published:** 2018-06-04

**Authors:** Wenxian Liang, Xiaoxing Zou, Rebeca Carballar-Lejarazú, Lingjiao Wu, Weihong Sun, Xueyuan Yuan, Songqing Wu, Pengfei Li, Hui Ding, Lin Ni, Wei Huang, Shuangquan Zou

**Affiliations:** 1 College of Forestry, Fujian Agriculture and Forestry University, Fuzhou, China; 2 Fujian Colleges and Universities Engineering Research Institute of Conservation and Utilization of Natural Bioresources, Fujian Agriculture and Forestry University, Fuzhou, China; 3 Department of Microbiology and Molecular Genetics, University of California, Irvine, USA; 4 College of Plant Protection, Fujian Agriculture and Forestry University, Fuzhou, China; 5 College of Life Sciences, Fujian Agriculture and Forestry University, Fuzhou, China

**Keywords:** *Euscaphis konishii* Hayata, Reference gene, qRT-PCR, Transcriptome, Gene expression, Normalization, *EkCAD1* gene

## Abstract

**Background:**

Quantitative real-time reverse transcription-polymerase chain reaction has been widely used in gene expression analysis, however, to have reliable and accurate results, reference genes are necessary to normalize gene expression under different experimental conditions. Several reliable reference genes have been reported in plants of Traditional Chinese Medicine, but none have been identified for *Euscaphis konishii* Hayata.

**Results:**

In this study, 12 candidate reference genes, including 3 common housekeeping genes and 9 novel genes based on *E. konishii* Hayata transcriptome data were selected and analyzed in different tissues (root, branch, leaf, capsule and seed), capsule and seed development stages. Expression stability was calculated using geNorm and NormFinder, the minimal number of reference genes required for accurate normalization was calculated by Vn/Vn + 1 using geNorm. *EkEEF*-*5A*-*1* and *EkADF2* were the two most stable reference genes for all samples, while *EkGSTU1* and *EkGAPDH* were the most stable reference genes for tissue samples. For seed development stages, *EkGAPDH* and *EkEEF*-*5A*-*1* were the most stable genes, whereas *EkGSTU1* and *EkGAPDH* were identified as the two most stable genes in the capsule development stages. Two reference genes were sufficient to normalize gene expression across all sample sets.

**Conclusion:**

Results of this study revealed that suitable reference genes should be selected for different experimental samples, and not all the common reference genes are suitable for different tissue samples and/or experimental conditions. In this study, we present the first data of reference genes selection for *E. konishii* Hayata based on transcriptome data, our data will facilitate further studies in molecular biology and gene function on *E. konishii* Hayata and other closely related species.

**Electronic supplementary material:**

The online version of this article (10.1186/s13007-018-0311-x) contains supplementary material, which is available to authorized users.

## Background

Quantitative real-time reverse transcription-polymerase chain reaction (qRT-PCR) has become one of the most powerful tools to study gene expression due to its high sensitivity, accuracy and specificity [1]. However, to get accurate and reliable results, a reference gene is necessary to normalize gene expression and avoid errors caused by different experimental procedure, such as sample amounts, quality and quantity of RNA, efficiency of enzymatic reaction and PCR efficiency [2, 3].

Most of the commonly used reference genes are housekeeping genes, such as actin (*ACT*), tubulin (*TUB*), polyubiquitin (*BUQ*), elongation factor 1-α (*EF1*-*α*), glyceraldehyde-3-phosphate dehydrogenase (*GAPDH*) and ribosomal RNAs (*18S rRNA* or *28S rRNA*). However, some data showed that expression levels of these housekeeping genes can vary considerably under different experimental conditions [4, 5], and also, in non-model plant species, usually the used reference genes are identified by the orthologous sequence of common housekeeping genes reported in model plant species due to the lack of genetic and sequence genome information [6]. Consequently, the unsuitable use of traditional housekeeping genes as reference gene in non-model plants can cause bias. Therefore, it is important to select proper reference genes according to experimental conditions [7]. Moreover, statistical software, including geNorm, BestKeeper, NormFinder and RefFinder, have been widely used as efficient tools to evaluate gene expression stability for qRT-PCR normalization [8–10]. Reference gene validation has been done in many plant species, such as banana [11], peach [12], soybean [13], amorphophallus [14], *Jatropha curcas* [15], *Isatis indigotica* Fort. [16], *Achyranthes bidentata* Blume [17], Kentucky bluegrass [18], *Salix matsudana* [19], *Rhododendron molle* G. Don [20], *Sapium sebiferum* [21], *Petroselinum crispum* [22], *Lilium* spp. [23], *Hibiscus cannabinus* L. [24] and *Dendrobium officinale* [25].

*Euscaphis* is a member of the family Staphyleaceae, which has two species in China: *E. japonica* Dippel and *E. konishii* Hayata. *Euscaphis* has been widely used in traditional Chinese medicine. Several chemical compounds have been isolated from *Euscaphis*, such as triterpene compounds [26–29], phenolic acid compounds [30, 31], flavonoid compounds [27, 31] and others [31–33], however, no molecular and gene expression data has been reported in *Euscaphis*.

Twelve genes (*EkUBC*, *EkF*-*ACP*, *EkARP7*, *EkEF2*, *EkACT*, *EkGAPDH*, *EkEEF*-*5A*-*1*, *EkADF2*, *EkTUB*, *EkPLAC8*, *EkLPP*, *EkGSTU1*) were selected as candidate genes according to transcriptome data from our lab (Liang et al., College of Forestry, Fujian Agriculture and Forestry University) (unpublished data), and their expression stability was evaluated by qRT-PCR across different experimental conditions: including five tissues (root, branch, seed, leaf and capsule), six different developmental stages of seed and six different development stages of capsule. Their expression stability was calculated using geNorm and NormFinder. Additionally, in order to validate our results, the expression levels of *EkCAD1* in different tissues were normalized by the most and least stable genes.

## Methods

### Plant material

*Euscaphis konishii* Hayata tissues were collected from Fujian Agriculture and Forestry University, Fujian Province, China. Tissues (leaf, capsule, seed, root and branch) were collected on November 15th 2016, and six developmental stages of capsule and seed were collected once every 15 days after formation. All samples were harvested, washed and surface dried and then frozen in liquid nitrogen and immediately stored at − 80 °C until required for further analyzes. Three biological replicates for each sample were used for RNA extraction.

### RNA isolation and cDNA synthesis

Total RNA was extracted from each sample using the RNAprepPure Plant Kit DP441 (Tiangen Biothch CO., LTD, Beijing, China), according to the manufacturer’s instructions. RNA was treated with DNase I (Tiangen, Beijing, China) to eliminate DNA contamination. RNA quality was determined by 1.2% agarose gel electrophoresis. The concentration and purity of total RNA was determined using a NanoDrop 2000c Spectrophotometer (Thermo Scientific, US). The A_260_/A_280_ ratio of total RNA between 1.90 and 2.10 was considered to meet the required quality for further experiments. First-strand of cDNA was synthesized using the First Strand cDNA Synthesis Kit (Roche, Switzerland) using 1.0 μg of total RNA in a 20 μL reaction volume according to the manufacturer’s protocols.

### Selection of candidate reference genes and primer design

Based on transcriptome sequencing data from our laboratory, 12 reference genes were selected to normalize and validate qRT-PCR experiments by screening for genes with relatively stable expression (based on their RPKM and fold change values), including nine novel genes and three common housekeeping genes. Their sequence/alignment/phylogenetic data are shown in Additional files 1 and 2. Forward and reverse primers of all candidate reference genes were designed using Primer Premier 5.0 with the following parameters: Tm values ranging from 50 to 70 °C, GC percent of 45–50%, primer lengths of 18–25 bp and product length of 90–200 bp. All primers were synthesized by Sangon Biotech Co., Ltd (Shanghai, China). Primer details are shown in Table 1.

**Table 1:** Primers used for qRT-PCR normalization

Gene abbreviation	Gene name	Primer sequence (5′–3′)	Amplicon length (bp)	Primers Tm (°C)	E (%)	R^2^
*EkUBC*	*E. konishii* Ubiquitin-conjugating enzyme E2-17 kDa	For: TCTGCAGGTCCTTCAATTCC	100	54.8/54.8	97.89	0.9998
		Rev: CGCAAACCCTAGAGAGAGTAAG				
*EkF*-*ACP*	*E. konishii* F-actin capping protein alpha subunit	For: CCAGTAACTCGCACCCTATTT	96	54.44/54.56	99.59	0.9994
		Rev: TCACTGTCACTTTCCGATTCC				
*EkARP7*	*E. konishii* Actin-related protein 7	For: CCTTCATTACCCATCTCCCATC	100	55.03/53.41	99.35	0.9878
		Rev: CTAATGAATCCTCGTATGACTGGAT				
*EkEF2*	*E. konishii* Elongation factor 2	For: GAGAGCGACAAGGGAATGAG	108	55.7/54.8	100.09	0.9997
		Rev: TATTACTGATGGTGCGCTGG				
*EkACT*	*E. konishii* Actin	For: CATTGTGAGCAACTGGGATG	125	54.01/54.21	103.21	0.9998
		Rev: GATTAGCCTTCGGGTTGAGA				
*EkGAPDH*	*E. konishii* Glyceraldehyde-3-phosphate dehydrogenase	For: TGGCTTTCCGTGTTCCTACT	113	56.14/57.12	101.1	0.9795
		Rev: TCCCTCTGACTCCTCCTTGA				
*EkEEF*-*5A*-*1*	*E. konishii* Eukaryotic elongation factor 5A-1	For: TCCGACATAGCTCCGATTCA	101	55.42/55.4	98.46	0.9991
		Rev: GAAGAGACGGAGAGGAGAGATT				
*EkADF2*	*E. konishii* Actin-depolymerizing factor 2	For: CCGAAGAGAATGTCCAGAAGAG	98	54.97/54.48	99.89	0.9998
		Rev: GTCCTTTGAGCTCGCATAGAT				
*EkTUB*	*E. konishii* β-Tubulin	For: AAAGATGAGCACCAAGGAGGT	108	56.18/55.60	98.69	0.9879
		Rev: TCACACACGCTGGATTTCAC				
*EkPLAC8*	*E. konishii* PLAC8 family protein isoform 2	For: GGGAATCGGAGGTAAAGATCAA	102	54/54	99.00	0.9822
		Rev: TGGATCTGAAGAAATGGGAGAC				
*EkLPP*	*E. konishii* Lonprotease-2-like protein	For: TTGGCCTCATCTATTGCTACTG	98	54.3/55.4	101.00	0.9931
		Rev: GTTCTCCTGTGCCCTCTAATG				
*EkGSTU1*	*E. konishii* Glutathione-*S*-transferase tau 1	For: GCCCTCATCCCAAACATACT	113	54.6/54	98.99	0.9999
		Rev: GAGATTGTTTGCAGCGAATAGG				
*EkCAD1*	*E. konishii* Cinnamyl alcohol dehydrogenase 1	For: GTGGGCTTTCCGTCAGTGTA	123	59.97/59.97	99.23	0.9969
		Rev: GGTCGGAGTTGGAGCTATCG				

qRT-PCR analysis for each candidate reference gene was performed on a 7500 Fast ABI Real-time PCR system (Applied Biosystems, US) using FastStart Universal SYBR Green Master (Roche, Switzerland). A 20 μL reaction mixture contained: 10 μL 2 × SYBR Green Master, 0.4 μL forward primer (10 μM), 0.4 μL reverse primer (10 μM), 2 μL cDNA and 7.2 μL dd H_2_O in a 96-well plates. The amplification conditions were as follows: 50 °C for 2 min, 95 °C for 10 min, 40 cycles of 95 °C for 15 s and 60 °C for 30 s. Melting curve was analyzed to determine primer specificity.

All samples were analyzed in three biological and technical replicates. Serial tenfold dilutions of cDNA template were used to generate slope of the standard curve to calculate amplification efficiency and correlation coefficient of each candidate reference gene.

### Data analysis

NormFinder and geNorm were used to analyze the stability of the 12 candidate reference genes under different conditions. Expression levels of each reference gene were shown by Cq values. Before using the two softwares, the raw Cq values was used to calculate relative quantities by the equation: Q = 2^−(sampleCq-mimCq)^. The values of stability (*M*) and pairwise variation (V) between genes was generated by geNorm, the lower *M* value is the gene expression is more stable [8, 34, 35]. Furthermore, the normalization factor generated by computing the pairwise variation of the two normalization factor was used to determine the most suitable numbers of reference genes with a cut-off value of 0.15 [17]. NormFinder was used to evaluate the stability of candidate genes by intra- and inter- group variations. The more stable reference gene will have lower stability value and inter- and intra-group variation.

### Validation of the candidate reference genes

In order to verify the results of our experiments, the most stable and unstable reference genes were selected to validate the expression of the *E. konishii Cinnamyl alcohol dehydrogenase 1* (*EkCAD1*) gene in different tissue samples (root, branch, capsule, seed and leaf). *CAD1* belongs to *CAD* family, which catalyzes the reduction of *p*-coumaricaldehyde, coniferyl aldehyde and sinapyl aldehyde to their alcohol derivatives which are then polymerized into lignin [36], *CAD* is one of the most used genes to manipulate to obtain plants with low lignin content [37]. qRT-PCR experimental method was the same as described above, and the relative expression level was calculated by 2^−ΔΔct^ method [12]. Data from three biological replicates were analyzed using analysis of variance (ANOVA) followed by Student’s t test (P < 0.05).

## Results

### Primer specificity and PCR amplification efficiency

A total of 12 candidate reference genes, including three common housekeeping genes and nine novel genes from transcriptome sequencing data of *E. konishii* were selected for qRT-PCR normalization. The details of gene names, abbreviation, accession number, primer sequence, primers Tm, product length, amplification efficiency and correlation coefficient are shown in Table 1. The specificity for each primer set was validated by melting curve. For all primer sets the melting curve showed a single amplification peak (Additional file 3). qRT-PCR efficiency for all 12 candidate reference genes ranged from 97.89% for *EkUBC* to 103.21% for *EkACT*, and correlation coefficients varied from 0.9795 to 0.9999 (Table 1).

### Cq values of candidate reference genes

**Fig. 1:**
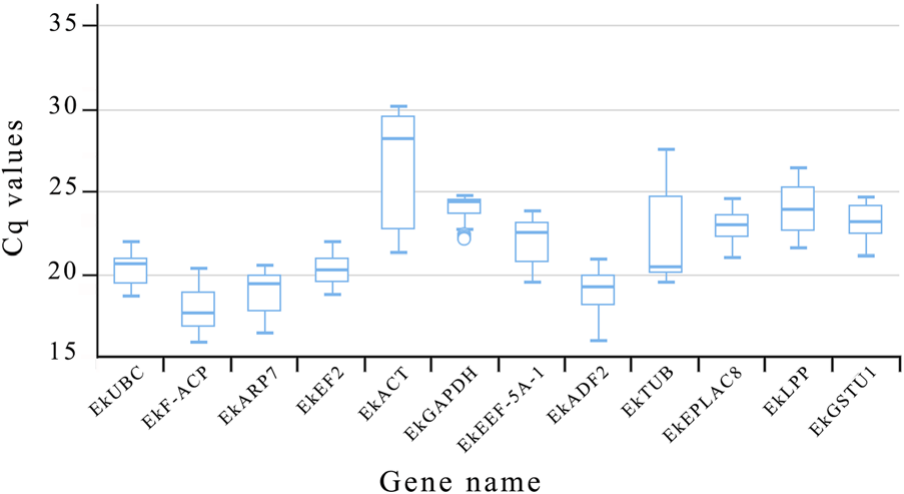
Cq values of the twelve candidate reference genes. The lines across the box indicate median values, boxes depict 25/75 percentiles. Whisker caps indicate the minimum and maximum values

Cq values for all 12 reference genes are shown in Fig. 1. The Cq values varied from 15.812 (*EkF*-*ACP*) to 30.121 (*EkACT*) across all samples, and mean Cq ranged from 18.0575 (*EkF*-*ACP*) to 25.6685 (*EkACT*). Moreover, *EkACT* expression levels were the most variable with 8.905 Cq, while *EkGAPDH* showed the least variable levels with 2.609 Cq. Since gene expression levels are negatively correlated to Cq values, *EkF*-*ACP* had high expression and *EkACT* with low expression.

### Expression stability of candidate reference genes

Expression stability of the 12 reference genes was analyzed by geNorm and NormFinder. Samples were divided into three different experimental groups: (1) five tissues (root, leaf, branch, seed and capsule), (2) six seed developmental stages and (3) six capsule developmental stages.

#### geNorm analysis

**Fig. 2:**
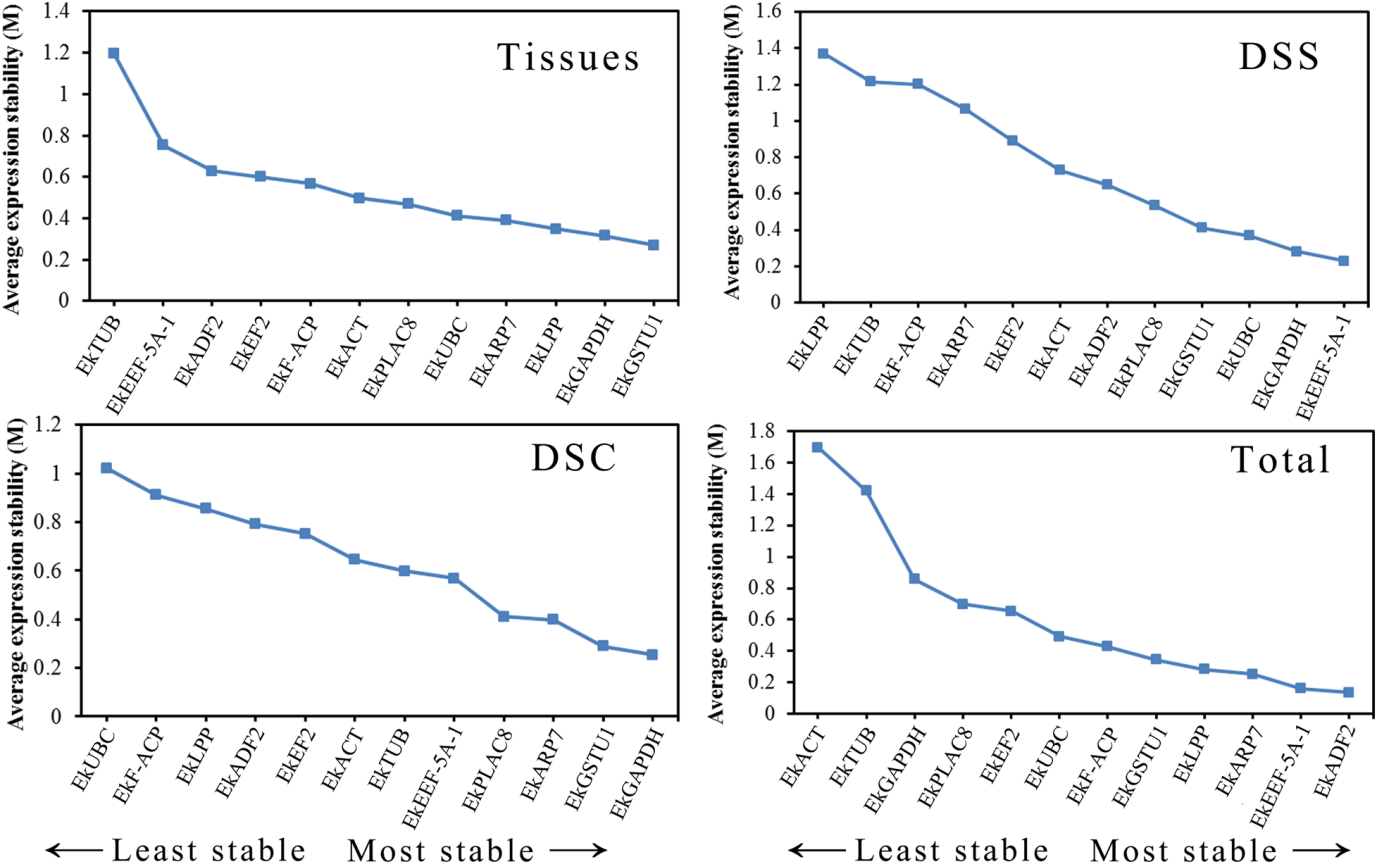
Average expression stability (*M*-value) of 12 candidate genes calculated by geNorm and ranking of the candidate reference genes in different experimental group. Tissues: five tissues sample sets; DSS: seed development stages; DSC: capsule development stages. Total: all samples

**Fig. 3:**
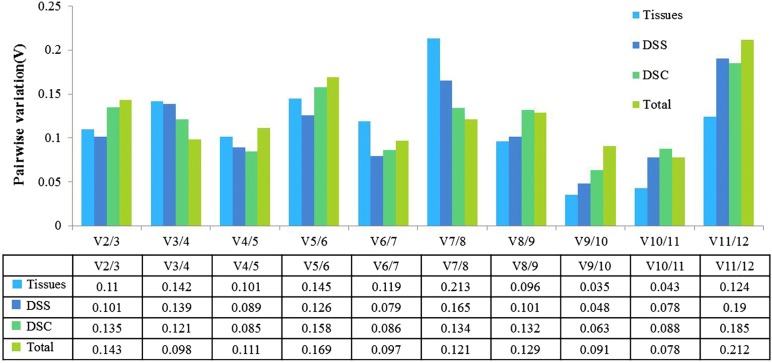
Optimal number of reference genes in different experimental groups using the geNorm. Pairwise variation (Vn/Vn + 1) analysis between normalization factors (NFn and NFn + 1) to calculate the number of reference genes in each experimental group. Tissues: five tissues sample sets; DSS: seed development stages; DSC: capsule development stages. Total: all samples

Gene expression stability was determined by *M*-value in geNorm analysis, the lower the *M* value is, the more gene expression stability. For the tissue group the two most stable genes were *EkGSTU1* and *EkGAPDH* with the lowest *M* value, and *EkTUB* was the most unstable gene. In the seed group *EkEEF*-*5A*-*1* and *EkGAPDH* were the two most stable genes through all the different developmental stages, and *EkLPP* was the most unstable gene. Finally, in the capsule group *EkGAPDH* was the most stable gene, followed by *EkGSTU1*, and *EkF*-*ACP* and *EkUBC* were the least stable genes (Table 2; Fig. 2). For all sample sets *EkADF2* and *EkEEF*-*5A*-*1* were the most stable genes, and *EkF*-*ACP* and *EkUBC* were the least stable. The minimum number of genes required for normalization in all the different groups was calculated by geNorm. The V2/3 values for all different experimental groups were below the cut-off value of 0.15 (0.143 of all samples, 0.11 for tissues samples, 0.101 for seed development stages and 0.135 for capsule development stages), which indicate that two reference genes are enough to normalize gene expression data (Fig. 3).

**Table 2:** Gene expression stability across sample sets calculated by geNorm

Gene name	Different tissues	Seed development stages	Capsule development stages	Total
*EkUBC*	0.412 (5)	0.369 (3)	1.023 (12)	0.491 (7)
*EkF*-*ACP*	0.568 (8)	1.201 (10)	0.911 (11)	0.428 (6)
*EkARP7*	0.390 (4)	1.065 (9)	0.398 (3)	0.251 (3)
*EkEF2*	0.599 (9)	0.890 (8)	0.753 (8)	0.655 (8)
*EkACT*	0.498 (7)	0.729 (7)	0.646 (7)	1.698 (12)
*EkGAPDH*	0.315 (2)	0.283 (2)	0.254 (1)	0.858 (10)
*EkEEF*-*5A*-*1*	0.752 (11)	0.231 (1)	0.568 (5)	0.159 (2)
*EkADF2*	0.629 (10)	0.649 (6)	0.792 (9)	0.134 (1)
*EkTUB*	1.198 (12)	1.216 (11)	0.599 (6)	1.421 (11)
*EkPLAC8*	0.469 (6)	0.534 (5)	0.412 (4)	0.699 (9)
*EkLPP*	0.348 (3)	1.368 (12)	0.855 (10)	0.284 (4)
*EkGSTU1*	0.269 (1)	0.412 (4)	0.289 (2)	0.344 (5)

#### NormFinder analysis

Expression stability values analyzed by NormFinder are shown in Table 3. For tissue group, *EkGSTU1* and *EkGAPDH* were the most stable reference genes, and *EkTUB* was the least stable gene, same as shown by geNorm analysis. In the seed group *EkEEF*-*5A*-*1* and *EkGAPDH* were the most stable reference genes, while *EkARP7* was the least stable gene. In the capsule group, *EkGAPDH* and *EkGSTU1* got the top rank, while *EkUBC* and *EkF*-*ACP* were ranked at the lowest. In general, the ranking was same as geNorm analysis (Table 3).

**Table 3:** Gene expression stability across sample sets calculated by NormFinder

Gene name	Different tissues	Seed development stages	Capsule development stages	Total
*EkUBC*	0.268 (6)	0.239 (3)	0.391 (11)	0.274 (8)
*EkF*-*ACP*	0.331 (7)	0.392 (10)	0.414 (12)	0.201 (6)
*EkARP7*	0.256 (3)	0.601 (12)	0.178 (3)	0.103 (4)
*EkEF2*	0.367 (9)	0.521 (11)	0.369 (8)	0.348 (9)
*EkACT*	0.546 (11)	0.379 (9)	0.365 (7)	1.495 (12)
*EkGAPDH*	0.240 (2)	0.171 (2)	0.102 (1)	0.493 (10)
*EkEEF*-*5A*-*1*	0.338 (8)	0.153 (1)	0.295 (6)	0.090 (2)
*EkADF2*	0.458 (10)	0.358 (8)	0.384 (10)	0.035 (1)
*EkTUB*	0.806 (12)	0.349 (7)	0.286 (5)	1.131 (11)
*EkPLAC8*	0.261 (5)	0.273 (4)	0.251 (4)	0.259 (7)
*EkLPP*	0.256 (3)	0.302 (6)	0.371 (9)	0.102 (3)
*EkGSTU1*	0.165 (1)	0.285 (5)	0.116 (2)	0.159 (5)

### *EkCAD1* expression and validation of *EkGSTU1* and *EkGAPDH*

In order to verify the reliability of the selected reference genes, expression profiles of *EkCAD1* gene was determined in different tissues. Relative expression levels were normalized using the two most stable reference genes (*EkGSTU1* and *EkGAPDH*) and the least stable reference gene (*EkTUB*).

**Fig. 4:**
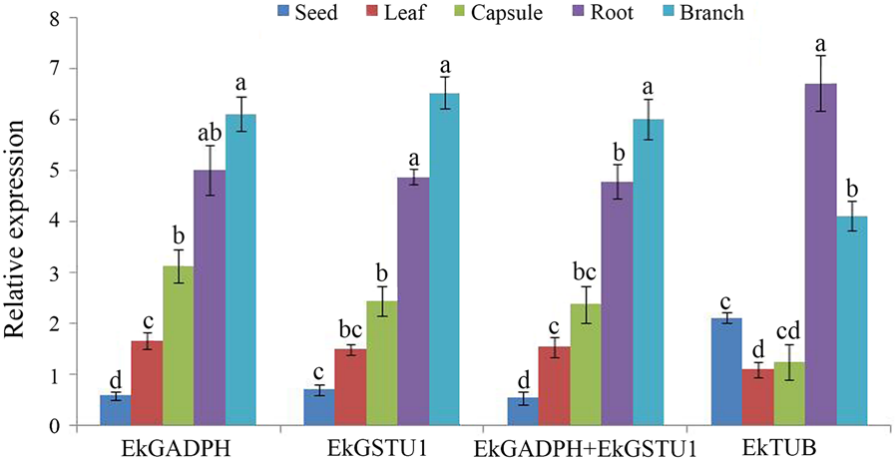
Relative expression of *EkCAD1* in different tissues. *EkGSTU1*, *EkGAPDH* and *EkGSTU1 *+ *EkGAPDH* were used as one or two most stable reference genes, *EkTUB* was used as the least stable reference gene. Data are represented as mean ± SD, different words indicate significant difference of the expression of the target gene based on three biological replications (P < 0.05, t test; n  =  3)

*EkCAD1* showed similar expression levels when single or a combination of reference genes (*EkGSTU1* and *EkGAPDH*) were used to normalize the expression. *EkCAD1* expression was up regulated in all the tissues except in seed. However, when *EkTUB* was used for normalization (unstable gene), relative expression profile of *EkCAD1* was different when compared when the normalization expression was done using the two most stable reference genes identified in our study (*EkGSTU1* and *EkGAPDH*) (Fig. 4). Our results suggest that the expression patterns of target genes are differed when normalized by different reference genes.

## Discussion

qRT-PCR is one of the most commonly used technique to determine gene expression in plants. To ensure the accuracy and reliability of the results, a suitable reference gene is necessary for data normalization. Conventionally, some housekeeping genes such as *ACT*, *GAPDH*, *TUB*, have been used as reference genes, however, no single gene can be used for all plant species, experimental conditions and/or tissues. Therefore, it is required to select proper reference gene(s) for certain species under different conditions rather than using common reference genes.

The development of high-throughput sequencing technology provides a more efficient approach to study plant molecular biology, and it has been widely used in plant genomes [38–43], plant transcriptome [44–47], plant ncRNA [48–50], moreover, the generation of large-scale gene segments and gene expression data by sequencing provides a new resource for the identification of reference genes, especially in non-model species [51–53]. Therefore, transcriptome data on *E. konishii* Hayata, available in our laboratory can be used as a tool to identify candidate reference genes. Asystematic study of 12 candidate reference genes in three conditions was carried in this paper, and their expression stability was calculated using geNorm and NormFinder.

*ACT* and *TUB,* the most widely used reference genes, did not show a good expression stability in *E. konishii* Hayata across all sample sets (Tables 2, 3). The phenomenon that expression levels of common reference genes varied in a large range has been reported in several papers [54, 55]. *GAPDH*, a common housekeeping gene also, has been widely used as reference gene in different species and experimental conditions [51, 56–60], in our experiments this gene was one of the two most stable genes in tissue sample set and capsule development stages, but it did not perform well in across all the sample and seed sets. The different performance of *EkGAPDH* in different experimental conditions in this study demonstrated that there is no single reference gene that can be used for all species or different experimental conditions [61–65].

In this study, *EkGSTU1* (glutathione-*S*-transferase tau 1), which belongs to tau class of glutathione transferases (*GSTs*) [66], was the one of two most stable genes in tissues sample and capsule development stages. *EkADF2* and *EkEEF*-*5A*-*1* were the two most stable genes in total sample set, *ADF* (actin-depolymerizing factor) play important roles in several cellular processes that require cytoskeletal rearrangements, such as cell migration, chromosome introgression, cleavage plane orientation and furrow formation [67–69]. *VvADF* has been identified as candidate reference gene for grapevine during anthesis [6], rubber tree duration of latex flow [70] and *TrADF3* was selected as reference gene in staminate and perfect flowers of *T. rupestris* [71].

It has been widely accepted that using combination of multiple reference genes to normalize gene expression can give more accurate and reliable expression patterns than using a single gene in qRT-PCR analysis [57]. Based on validation results of target gene expression, when *EkGAPDH* and *EkGSTU1* were selected as reference genes for normalization either single or combination, the target gene *EkCAD1* showed the similar expression pattern among different tissues, which indicated that the expression pattern of *EkCAD1* was nearly identical when normalized with a single reference gene or two. Interestingly, in the tissue group, the combination of traditional housekeeping gene (*EkGAPDH*) and a novel identified reference gene (*EkGSTU1*) were identified as the most stable reference genes, suggesting that combination of traditional housekeeping genes and newly identified reference genes based on transcriptome data can be used as a good strategy for expression normalization of *E. konishii* Hayata genes.

## Conclusion

In this study, we evaluated the expression stability of twelve candidate reference genes, including three traditional housekeeping genes and nine novel genes based on transcriptome data of *E. konishii* Hayata. Additionally, the expression pattern of target gene *EkCAD1* was determined in different tissues to further verify the reliability of the identified stable reference genes. This study shows the first data for reference genes validation on *E. konishii* Hayata. Our study will contribute in future studies of gene expression in *E. konishii* Hayata and related species.

## Additional files

**Additional file 1.** Sequences of 12 candidate genes and 1 validation gene.

**Additional file 2.** Alignment and phylogenetic tree of 12 candidate genes and 1 validation gene.

**Additional file 3.** Melting curves for the 12 candidate reference genes.
