# Supplementary material for: Selection and evaluation of reference genes for qRT-PCR analysis in Euscaphis konishii Hayata based on transcriptome data
Source: Plant Methods. 2018 Jun 4;14:42. doi: 10.1186/s13007-018-0311-x (PMC5985561; doi:10.1186/s13007-018-0311-x)
Supplement: Supplementary file 1 — Additional file 1. Sequences of 12 candidate genes and 1 validation gene. [file 13007_2018_311_MOESM1_ESM.docx]

| >*E. konishii* Ubiquitin-conjugating enzyme E2-17 kDa |
| --- |

ATTGCTGTTTATATTAGGGTGAAAAACTTTGGTACGAAATGAGACCTTAGGTGGCTTGAATGGGTAATCTGGTGGGAAATGAATGGATACAAGGAACACACCCCCAGAAAATGGGCTGTCAGATGGGCCCATAATGGTTGCTTGCCAGTGAAACATGTCATCAGCAACAGGGCCGGCGCTGCAGGAAGCAGGAGGGTCCTTCTGCAGGTCCTTCAATTCCTTGTTGATTCGTTTTGAAGCCATCACAAACTCTAGAGTTTCAAACTCGTATTAAGTTCCTTACTCTCTCTAGGGTTTGCGTCGAAGTTTCGACTTCGATGAAGTCCCAGAGTCGAAGTGTGAGGGCGTGTATGAATTTTAATGGGTGTGTGTGGAGGCACCCCGTATCTGTGATACTGGTGGCGGTGAGTC

>*E. konishii* F-actin capping protein alpha subunit

ACTACGTCTACACCACCGCTCAAGATCTTTTTTCGCTCACACACACCAAAGCTTTTGTAGAACACACAGCATTCTCTCTGTGTATGTAGTTCCTCTCGAAACGGCTGCTTCAATGTCAAGTATGGAGAGAGGTAGAGCCATGGCGGACGAAGAACCAGAGCTCAACGCCGACCAAAAGAAAGAGATCGTCAAATGGTTCCTCCTCAACTCCCCTGCTGGTGAAATCCAATACGTTGCCAAAGATGTAAGGTCGGTTTTGAATGACGAAGATGTGTATCAAGATGCGGCGTCAGAGGCATTTCCTTTGTACAATAAGACTCACGCGATTTGCCTCGAGATGTCCGACAGAAGCGGCGATGTGCTAATTACATCACATGGTGAGCTTGATAAGAATGAGTTCCTTGACCCCAGGACTGCCCAAGTTGCTATAGTTGACCATGTAAAACAAGTTTGTACAGAGGTGAGACCTGCTGCTGATGAGGAACTTCCATCTCCATATATTGAAGAATTTCGATGTGCTCTGGATGCAGAAATTCTCAAATATGTGGGTGAAGCTTATCCGAAAGGTGTCTGTTCAGTTTACTGTACAAATGGGAAAGATGTTGAGGAACCTGGATCCAATTTCGAGCTTGTTGTAGTGATTTCTGCTGCTAGACATAGCCCCCAGAATTTCTGTAATGGAAGTTGGCGTTCAGTATGGATCATTGAGTTCAAGGATGATTTACAAATACTAGATTTGAAAGGAAAAGTTCAGGTGGGTGCCCATTATTTTGAAGAGGGAAATGTGCAGTTAGATGCAAAACATGAATGCAAAGATTCAACAATTTTTCAGTCCCCTGATGATTGTGCAATTTCTATAGCAAACATTATTCGCCACCATGAGACAGAGTACCTGGCATCTCTCGAGGCATCCTATTCAAATCTGCCAGATACCACTTTCAAGGATCTTCGGAGGAAGCTTCCAGTAACTCGCACCCTATTTCCATGGCATAGTACTTTGCAATTCAGCCTGACAAGAGACATCACAAAAGAACTTGGAATCGGAAAGTGACAGTGACTTCCTCTATGTGTTGGTTGGCCCATTTGTATTTTGAGTATGTGAATCCTTCAAGAGTCCATTTTTAGTCATTAATTTGCTGCGAGATTCCTATGTGAATCCTTCAGAGAGTCCATTTTTAGTCATTAATTTGCTGCGAGATTCCTATGTGAATCCTTCAGAGAGTCCATTTTTAGTCATTAATTTGCTGCGAGATTCCTTGTGCCTATAGAAATTTTCTTCCATTTTCAGTTTTTGGGTATTGCCTTGTGTAGAGTTGCCTTGTGTAGAGTGAAGAGTTGTGGATGCTTTAATTTTCTTGTCATTATTATGGAAAATTTTGGCAAGTTTGTAATCCTACTATATGCTCTGGGAACTGGGTGTAATACAAAAATCATTTGGAGACAGCATTGCATGTAACAACTTTCCCAGAGATGGATAAAGCCTATTCTGGACTGCTGGTAATTTTGTGCCGGAGTCTGTATGTTCTTGACAGAAGTGCGCCTTATAAGATATTTTCTGTAGCCCACTAAGACTTAAATAATTACTTATATATTTCAAAGTATTATTCAAAACTCAGACGTATTTCACGGTTTGTTTCTAGCTCAATTTTTATGTGG

>*E. konishii* Actin-related protein 7

TGGGTGTTTTTTTTTTTTTTTTTTGGGATAAGTTACCAAATGATTTTCTAACTACATGGAGTCCTCTATCAATTCATGACTTTCCACTACCATTCTGAACCATTATATTACAATGGATAATGGAACTAAGGAAAATAATGAACTCACTGAGATGAGAATAATCAGAAGCATTTCTTGTGAACAATGGAAGGCCCAGTTTCATCGTAGTCTGCCTTGGTTATGTGCTGATTCTGAGGGAACACAACTTTGGCAAGTATTGCACCTCCAATCCATGCTGAATACATTGTCAAATTCTCTGGCATGTATTCTGGAGGCTTTACTAGAGAAGGCCGAATAGCGGATGAGCACAGGCTAGCTTCTTTTTGGAACCTATCTTCAAAACCAGTCATAGAAACAGTACCACCACAAAGTACAGTGTTTTCTAGCAGCTGCCGGTGATTTTCAGATGACACTGTTGAAATAGTACGAACAAGCTGCTCAACAATTCCATGTGCCTCTAAACCTAATATAGATGGTTGGAATAAAGCTTCACCAACAGTATATCTTTCTTTTCCGATTGTTATCACCTGTCCATCAGGAAGGGTATGCTGCTCTGTCTGGCATGACTGTTGGGTCTTATTATAAGCAAGTTCATCTTCTGCGCAACATGAATATTTCTCTTTCAATTGCTCAACATCAGAGACGTTGAGATTCAGCAGGGGATTGGATTTACCAAGTTCCTGAGCAAGTAACTTTGTCAAATCAACCCCTCCGATTTCAAATCTTCTTGAGGCAATGTGCTGAACGGCACCCTCGATTACTGGTGCAATATCTATCTTCCCATGACCGATATCAACAGTGCATCCAGAGATCCTTCCTACAGCATATAATGATAATACTGCTTGCTCTGATGCATAAAAGCCCGATATGTTAAATGTCTCAAACATCAATTGCACCAATTGTTCTCTAACAACCTTGGGGGTACAAAGTGGATCAGTAAATAATATTTGTCCTTCATTACCCATCTCCCATCCAAGGCCAGTATATAGAACATGGTCTAACAAGTCTTCTACTGCATCCCAATCTCTAATGAATCCTCGTATGACTGGATCAACAGTTAGATCCTCAAACAGTGAACTATCTGTCATTGATCCATCATCAAGTATGCGCTTCATTTGGGTAGGAATTATCATGGATGGAGCTTGATCCGGGATTGCCGCGCCTGCTTTGAGGAGCTTGGACCCCACATCGACCACCACAGCCTCCATTTGCTTCTTCTACGTCACAAAACCAAACCTAGTTTTAATATTTTACTCTCTGCAGAACCCTAGATTTTGGGTAGAACGGTTTCATAAACATCTCTAAAACCCTTCCGTTTTTTCGTTAATTTGTTCTCTG

>*E. konishii* Elongation factor 2

GTTATAATATAAGCAGTTCAAACAAACACGAACATCATCCACGGGAAAATTAAGCGGCCTGCAGCCGCGCAACAACGGCGATATGTCTTCAGCAGGAAACGGTGACGACGAATGAGGACGGGGAAGAAAAAAGCGAACAAGAAATCTCGCCTTGGCAGGCCGGCGGCGGGAAGAAGCTCAGAGCTTGTCCTCGTACTCGGACAGAGGGGTGATCTGCTCCTTGAGGCCCTTGCGCTTGCGGATGTCGGCGACGATTTCGGCGGCCGAGGTTCCGGGCTCGAGGGGGTCGGTGGAGAGAATGTCCCAATGGTCGAAGACGCACTGGGGGAAGGCCTGGCCCGACGTGGCGGCGCGGAGGGCCGTGGAGAACCCGAAGGACATGATGACGGGAAGGTAGGCCTTGATGTTGTAGAGTGGGGTCCCCGGGCGCTGGATCTCCTCGAAGACGTGGCCGCGCTTCTGGTTGAGCACGCCGTAGATGCCGCCGAGGGCCTGCTCCGGGGCCTGGATCTCGACGAGGTAGACGGGCTCGAGAAGGCGGGGCTTGGCGGTGAGCTGGGAGGCGTAGATGACGCGGCGTGCGGTGGGGATGACCTGGCCGCCGCCGCGGTGGATGGCATCGGCGTGGAGCACCACGTCGCAGACCTCGAAGCAGATGCCGCGCATGTTCTCCTCGGCGACGGCACCCTCCTTGGAGGCCCACTGGAAGCCGGCGACGACCGAGTCCTTGATCTCGTTGAGGTACTGCACTCCCTTGCACATGTCGACGACCATGTTGGGGCCGGTGGTCTCGGGGCCGAAGCACCAGATCTTCTTCGCCAGGTCCTTGTCCCAGCCGAACTCCTCCGCCAGGATCTTGGAGCGCACCTTGGGGTCGTCACGCGGCCCGATGCGGCCTTCGTCGATGGCCTCGGCGAGCCCCTCCTCCAGGGGCCGCGCCTCCATGTAGAGGCGGTTGTGCTTGTTGGGCGACTTGCTCATCACCGTGCGGCACGACTTCTCCAGCACCGTCTCGCGGAAGGACACCACCGGGTCCGACACGATGATCTCCGCTCCGCCCATGAAGTCCTCCTGTAGATCCTTGAGGCAGATCTCCAGGTGCAGCTCCCCCGCGCCCGCCACGATGTGCTCCCCAGACTCCTCGATCGTGCACACCACCATAGGGTCCGACTTGGCCAGCCGCTTAAGCCCCTCGACCAGCTTCGGCAGGTCCGAGGCCACCTTGCATTGCACCGCCACCCGCACCACCGGCGACACCGAGAACTTCATCGCGCGGATAGGGTGAGCGTCCACCTCCTTCTCGTTCGTCAGCGTCGCGTTCTTCGTGATGAACTGGTCCAGCCCCACCATAGCCACCGTGTTCCCGCATGGCACGTCCTCCACTGACTCCTGCCTCTTGCCCATCCAGATCACCGTCCTCTGCACGCTCTTCACGTACAGGTCCTTCTTCTGCCCTGGGACGTAGTTGGGCCCCATGATCCGCACCTTCATGCCAGTCGCCACCTTCCCGGCGAACACCCGCCCAAAGGCGAAGAACCGCCCCTTGTCAGAGGCCGGAATCATCTTCGACACGTAGAGCATCAGCGGCCCCTCTGGGTCGCAGTTACGGATCGCGTTGGCGTACATGTCGTCCAGCGGACCCTCGTACAGGTTCTCCACACGGTACCTCTGCGCCGTGGCCGGGGAAGGAAGATGGAAGATCATCATCTCCAGTAGTGCGTCCGCGGCGGGCAGCAACGTTTGCATGACGCGCTTCATCAGCACCTTGCCCGTCAGATCCTTCTCGTCCGACTTCATGGTCACCTTCAGCTTCTGGAGCATGGGCCACAGCTTATCCTTCTGATCGTTCATGCA

>*E. konishii* Actin

TGGAAAGCAGTCAAATGCTACTCGAAATTAATTAACAATGAAAGAATTGGGCCATTTTTGGCAATCAACTCCTCATAGTCCTCAGTTCCACATGAGAATAACATAGAAAGTAAAGAAAACTAGAATGTAAGTGTTGCAAGCTGCTGCATTTCTTTCCAATAATAAATGGAAGTACTTTCCTCCAAAATACTACTCTTGAACATTTAGAAGCATTTCCTATGAACGATTGATGGCCCGGACTCATCATACTCTGCTTTTGCAATCCACATCTGCTGAAAAGTGCTCAGGGATGCCAAGATGGAGCCCCCTATCCAGACACTGTACTTCCTCTCTGGTGGTGCCACTACCTTAATCTTCATGCTGGATGGGGCAAGGGCAGTGATTTCCTTGCTCATTCTATCAGCAATACCAGGGAACATGGTGGACCCACCA

>*E.konishii* Glyceraldehyde-3-phosphate dehydrogenase

ACGGCCAGTGGAAGCACCATGACATTAAGGTGAAGGACTCCAAGACCCTTCTCTTTGGTGAGAAGCCTGTCACTGTTTTTGGCGTCAGGAACCCAGAGGAGATCCCTTGGGGTGAGACTGGAGCTGAATATGTTGTCGAGTCCACTGGAGTTTTCACTGATAAAGACAAGGCCGCTGCCCACTTGAAGGGTGGTGCAAAGAAGGTTGTTATTTCTGCCCCAAGTAAGGATGCTCCAATGTTCGTTGTCGGTGTCAATGAGAAGGAGTACAAGTCAGATATTAACATTGTTTCAAACGCTAGCTGCACTACCAACTGTCTT

>*E.konishii* Eukaryotic elongation factor 5A-1 isoform 3

AAAAAAAAAAATAAAAGAGAACAAATATTCTCTCTTTTTTGTTACATTCCCAAAAACAATAAAGAATAAAATGACATTTCCAAATTTTTTATTAATAAACTTTCAAAATCCTCTTATTAATTTGAGACCCTTCAGTTAAACTCTTATCAGGTAAGTTTAATAAAATATGTAATCAGCTTTCCAGTAAACACCTAAATTCTGTATAAACTTATAATAATTTTATTTAATTTTTTGTGTCTATCTTTACAGTACGCACACAAGAAAACTAATTAAACAATACAAGATACATTTCTCAATTTGACAACTACAAATTAAGAAACAGAAATTGACATTTGAGACGAACCAGCCTTTATTCTGCAACGATTCTCATTCCAAATGTAATAAAGTCCTCAATATTAAAAGTTCAAATGCAATAACACGAACCAAAGACAGATTAAAACTGCAAGTTTCAAATGAAGGTGCCAAGAAGTGTATATACAGTCTTGCTTGGGGATAAAAATTCTCCACACCATAAACCAGGGCCTGGTTCAAGCAAAGCACAACTATTGATTATAAAAGGCTAATTAATAATGGAAAAGTTTCCTTATAGGTGGAGGTCACGTATCATCATCAAGATATTCCTGAAAATGCTGACCTTTGGTTCTCAAACCAACATAAAATCCAACTATTACTGTTTCGGTTTATCATTTCAAAGGCTTGAAAATTCAACATTAACAATGGCTAATTACTTTGGCCCAATGTCCTTGAGGGCACAGATCTGCTCCTCTCCCATAGCAGACATAACACTCACAACCAGATCTTTCCCCTCGGCAAACCCATCCTTGATCTGAGAAAGCAGACTGTCATCGGTTGGAAGCCTCAGGTCATCCTTTGTATTACCATTCTCAGTCAGCAGACTCACAAATCCATCCTCAGAAATATCAATCAGCTGGTAGTCAGTACGGTTGACATGGGGAACATCACAGTTGTGGGACGAGGGAACAATATCCTCAAGCTTTTTGGCAGTGAAGATATCAATTCCAACAAAGTGGCATTTAGCATGACCATGCTTGCCAGTTTTTGAAGTTGAAACCTCAACAACCTTGCAAGGGCGGTTCTTGATGACAATGTAACCGTTCTTGCGGATGGTGCCGGCCTGCTGAGGGTAGGTCTTGGAAGCTCCGGCATCGGCCTTGGACTCGAAGTGGTGCTCCTCGTCCGACATAGCTCCGATTCACGAGAAAAAAAAAAAAATTACAGAGAGACGAGAAGTAGAGCTAGTGAGAGAGTGACGACGAAGAGACGGAGAGGAGAGATTTTATATTTATAGGGAAATCTTAAATTTACGGGACAAAAGGATTAGGAAAAGTGGGGGGTGGGGGCCTAGGGTTTTCTCTAATTTGGAATAATTGAAAGATTTTCCACCGTTGGATTTGAAAGAATTTGAACGGTGCGATTTCAGTTATTTTAGGGAGCAGCTTGGTCTGCGTGACAAGCACGCTGATGGCGTGAGTGGGAGCACGTGGGCGGCTTAGTTTAATTAGTCTTTTTGGTTTTGCTTGTTGCATGGGCCCAATTTTGGACCCAAATCGAATTTGAACCCCTTTTGTCGGTTGGGTTAATTGGATATGGATGCACCCCTGGATGTTATTTTTTCCGTATTTATTTATTATTTTGATCAGTATCCGTCTTTCTTTTAGAAAGCTATATAAATTGATACGTGCAATAACGTTTTATGATAGGTTCAAGGGTCCAATTCAATTTCCAAATCATTGTGGGTCAATTTGGCCATTTGTAGGTGAAATCTCGCACATGATGACCTAAATAATTCAAACAGTA

>*E.konishii* actin-depolymerizing factor 2

GGCGGGGGGAGGTGTGGAGCCACCGCGTGCTGGAAGTTCTTCATTGTATATGGAAGTCTGAGAGAGTCAATATCTCTATCTCCATCTATACGTACACCTATACATACACGCATCTTGTTCTTCGCCTTCTCCAGATTCGATCTACTCTATTCAGTCCCGTAGCTTCCGATCTGCGCAACGATGGCTAATGCAGCGTCGGGAATGGCTGTGCATGATGACTGCAAGCTCAAGTTTTTGGAGCTGAAGGCAAAAAGAACCTATCGCTTCATAGTGTACAAGATTGAGGAGAAGCAAAAGCAGGTCATTGTGGAAAAGGTCGGTGAACCAACTCAAAGCTATGAAGACTTTGCTGCAAGCCTTCCTGCTAATGAGTGCCGCTATGCTGTTTATGATTTTGACTTTGTGACCGAAGAGAATGTCCAGAAGAGCAGGATTTTTTTCATTGCATGGTCTCCTGATATATCAAGGGTTAGAAGCAAAATGATCTATGCGAGCTCAAAGGACAGGTTCAAGAGAGAACTGGATGGTATCCAGGTAGAGCTGCAGGCAACTGATCCAACTGAGATGGGTCTTGATGTTATTAGAAGCCGTGCCAGCTAAATACAACTCTCCAAGCCTCGGGGACGGAGAATATTATGCTATTTGCTTTGCTTTAAGTGAAATGTTAATAGTTTGTGCCTTGTTGGGAGGTGAACCTGGCAACTTATAATATTGGTTTGTTATCCATGTTTTACGGTATCTTACAGTATTGCCTTTGACTAGAACAAGTTCAGTGATTTTAATCATATCTTGCAATTTGTTGTGTTAGTTTGTGGATTTATGCAATGTCTTTTTTCGTATAGTTGTCTACAAGATTAACCATCTTCTGGTGTAGATAACACCATCTGTTTCTGTATTTAATATACCCTTTGTATTCTGTTTTTACTAGGTAGATTAAAAATGGGTATTGACCCTTGCCCTGTAGGACGTAAGCTATTGTACTTGATGAAAGAAAGGTGTATTTGATTTACTTTTACGCTGATATAAAATTTACTGAATTATTTTTGACTGGTAGGTTATTCAACACTAGTCTTAA

>*E.konishii* β-Tubulin

GCGGAGGCAGCAAGTAACCCCGCTCATTGTGGCAGAGATGAGGTGGTTGAGGTCGCCAAAGCTCGGGGTGGTGAGCTTCAGAGTTCGGAAGCAGATGTCGTAAAGGGCCTCATTGTCCAACACCATGCACTCATCGGCGTTCTCAACCAACTGATGCACCGAGAGTGTGGCATTGTACGGCTCGACCACGGTGTCCGACACCTTTGGGGAAGGAAAGACGGAGAACGTGAGCATCATGCGATCAGGGTACTCTTCCCTGATCTTCGAGATCAAAAGAGTTCCCATGCCTGACCCCGTTCCGCCACCCAGCGAGTGGCAGACCTGAAACCCTTGCAAGCAGTCA

>PLAC8 family protein isoform 2

TTCCACACAACCATTGAAAGTCGCACATTCATCACTGCATTCTAAATACATAATCAAATCCACATACATATAATGCATCATACGTACAACCTTAGAGTATTGTTCAACCCAAACAAAGTATTACAATATAGGAGTATTGTTCAACCAAACAAAGTGAAAGTTCTTCAAAAGCAGTTCATATACGGACATACAAGTTTTGGTCGATGATACAACGTATCACACTCAGAATTTGTATATAGCCAGCGTACCTTATTTCATGTCAAGGATCTTAGGCAGTACGGCCCATGGTCTGCTCCCCAGGAGGGATCATGACCAAAATTGGTTGAGCATTGAAACCGGGATGTGGGAGCTTACGGCGGAGTTCACGACCTTCCTGACAGAGTGCGCATGTGTGGCAAAAGACATGAGTTGCAAAGTCACATGCTGTCTCACATTGCTCACGTTGAACCTCTTCTTCCATAAAGCTTCCACAGCATCCACATGACCTATGGAGTGCCTCGCAGCTGCCCTCTAAGTTGAATTTCCGGCGGATCGCTGTACGGCTAGGATATGAAAACCAGGGTGCAAGACAGTTCCAACCAAAAAAGGAATTTCCAATCAGGTACAAGCCAGTGTAAGGCAAGCAGTGGTTTGCAAAAGTACCAGGACTAGATCCAATTCTCTCAACATTGCTTCCATACAGCACGCAGGGGGCCATGCTTCCAAGAAGACAAACTTCAAGATCGCTGCTACAAAACTCATCGTTGCGACCCAGGCAAGCGAACATGGAGGAGTCCCATTGGGCCCGACCTATCGGCTCCCCCACCACGCTCCCATGCCCTAGCGGCAAACCATTGGCAGTCCAGCCATACGCAGAACCTGGAGCCGGAGATTTCTCCTCCGCGTCCTTGGAGAAGGACTTATTATTCGCTTTCTTCTCCGAATCCTGCACCGGTTTGGGCAGCAAAGGGCTCGATTCCTGGTGATTGTTCCCGCTATCGGCCATTGGGGAATCGGAGGTAAAGATCAAGAACACTGCAACTGAATCGGTGATGAAGGAAGAGAGAATTGAGAAAGGAAAGATTGAGTGGATCTGAAGAAATGGGAGACTTTGAAAGGTCGTCCTCCTAAACCG

> *E.konishii* Lon protease-2-like protein

AGGAAGGAGAAATAGAAACGGGAAAGAGAGCCGTCGCCGTTCGGTTAAGTTCTGAAGAAGAGAGAGTACCAACCACGAAACCACCTCCGTATTCAAAAGCCTCTCCTCTCCCGTTTCTATTTCTCCTTCCTAGTCAACGGACATGGCCGAATCGGTGGAGCTACCGAGTCGCCTCGGGATTCTTCCCTTCAGGAACAAGGTCCTCTTGCCGGGCGCCATCATTCGAATTCGCTGCACTTCACCTAGCAGCGTCAAGCTGGTGGAGCAAGAGTTATGGCAGAGAGAAGAGAAGGGATTGATTGGGATTCTGCCTGTTCGTGATGCTGCTGAGTCAACAACTGTGGGCTCCCTTTTATCTCAAGGAACTGATTCTGGAGAAAGGAGCACAAAACTTCAAGTTGGTACATTAGATGGTCACAAACTTGATGGAAAAAATCAACAGGACATTATTCATTGGCATACTAGAGGTGTTGCTGCTCGTGCTTTACATTTGTCAAGAGGAGTGGAGAAACCAAGTGGGAGGGTCACATATACAGTTGTTCTTGAAGGCTTGTGCAGATTCAGTGTGCAGGAACTCAGTACTAGAGGAACATATTATACTGCACGAATTGTTCCTCTTGAGATGACAAAGACCGAGATGGAGCAAGTGGAGCAGGATCCTGATTTCATAACCTTGTCTCGCCAGTTCAAAGCAACTGCAACGGAGCTTATGTCTGTTCTCGAGCAGAAACAAAATACTGGTGGAAGGACAAAAGTTCTTTTGGAGACAGTTCCAGTTCACAAATTGGCAGACATATTTGTTGCTAGTTTTGAGATTAGTTTTGAAGAACAGTTATGTATGTTGGATTCAGTTGATCTAAAAGCAAGGCTCTCAAAAGCTACGGAATTAGTTGACAGGCATTTACAGTCAATACGTGTAGCAGAGAAGATTACACAAAAGGTTGAGGGACAGCTGTCAAAATCACAGAAAGAGTTTCTTTTGCGTCAGCAGATGAGGGCTATAAAAGAGGAGCTTGGTGACAATGATGATGATGAGGACGACCTGGTTGCCCTTGAAAGGAAGATGCAGAGTGCAGGAATGCCTTCAAACATCTGGAAGCATGCGCAGAGGGAGTTGAGGAGGCTTAAAAAAATGCAGCCTCAGCAGCCTGGATATAGTAGTTCACGTGTTTACCTGGAGCTTCTTGCTGATCTGCCCTGGCAGAATGTCACTGAAGAACAAGAATTGGACTTAAAGGCTTCAAAAGAGCAGCTTGACAGTGACCACTATGGTTTAGTCAAAGTCAAGCAAAGGATTATTGAATACCTAGCAGTTCGCAAGCTTAAACCTGATGCGAGAGGCCCAATTTTGTGCTTTGTTGGTCCTCCAGGTGTTGGGAAAACATCTTTGGCCTCATCTATTGCTACTGCTTTGGGTAGAAAATTTATACGCATATCCCTTGGTGGTGTTAAGGATGAGGCTGACATTAGAGGGCACAGGAGAACGTACATTGGAAGCATGCCTGGACGTCTTATTGACGGGTTAAAGGTTGCCGCATTTATGGATTTGCTGTTACTTCAAAATTTTGTTAATTTGTTTGATATTTGGTTCATCATATGCTTGAGGTGAGGTGGTGTTGGGTTAGATGGTCAAAAAAAAAAAAGTATAAAGCCAGGGAGTAGAATGTTTAAAATGTGACCACAAATTATTGTTGTTCTTTCTGACAAATACTCATTCTGTCATATCTCTTTATGCAAGTTATATTAGTTTGTGGCAGAACTGTTTGTGAATGCTGAAACTATTTAACTTGGACTATTTTTCCCTGCCAGCTGAATTTTAATTTCTGATGGGGGATTGGGAAAGTTTACAAAGTTAATTTGCTATTAGTGGAGCTTGATTTGTAGCTATTCCGTAAATCCTCTTCGTTGTTGAACAGTCAGATGATTTTTTGTGCTTCACAGTTAATTCTCTTGTTAAGCTAGGTTCTGTTCATGTAGCTCGAATTATAATTTTGTAATTAGCCCGCTATTGTCCTTAAATTGGGTGCATTTTCTTTGTATTTTCCTTGGCTTGCATTTTTATTAGTTGTATCTGCTTTACTGGTAGTATATTTACCTTGTAGTCATTTGAGCAACTGATATATGCTTTACACTTGATCATAGAGGATACTGTGCGTTAAATTTATGACTGGTTGTGTTACTGTTTTCTTTCCTTGTCTTCTTGAGTTTCAGCCTGGTCAAACCCCCACCACACACACCCACACACCCACTTACCCACCTGCACTCATCTTGGGGTTGAGGTGGACTAAATTGTTATTATGTATTCTGACTGTTAGAGAGTAGCTGTATGCAATCCAGTGATGCTACTGGATGAGATTGACAAGACGGGTTCTGATGTACGTGGTGATCCTGCTTCAGCTCTTCTGGAGGTTCTTGACCCTGAACAGAATAAAACATTCAATGATCACTATTTGAATGTTCCATTTGACCTTTCAAAGGTAGTTTTTGTGGCGACTGCAAATAGGGTTCAGCCTATTCCTCCACCACTCTTGGATAGGATGGAAGTCATTGAGCTGCCTGGATACACACCTGAAGAAAAGCTTAAAATAGCCATGCGACATCTAATTCCACGAGTTCTGGATCAACATGGCTTAAGTTCCGAGTTCCTTCAAATTCCAGAGGATATGGTCAAACTTGTAATTCAGAGGTACACTAGAGAGGCAGGTGTCCGGAATCTGGAAAGGAATTTGGCTGCTTTGGCCCGTGCAGCAGCAGTGAGAGTTGCAGAGCAAGAACAAGCTGTCCCACTGAGCAAAGATGTGCACCGGCTTGCTTCCCCGCTGCTGGACAACAGACTTGCTGATGGAGCTGAAGTCGAAATGGAAGTTATTCCAATGGATGTAAACAATCTTGAGATATCAAATACATTCAGAATTGCCTCGCCTTTGGTTGTAGATGAGGCTATGCTGGAAAAAGTACTGGGGCCTCCAAGGTATGATGACAGAGAAGCTGCCGAACGTGTTGTAACTCCCGGGATATCTGTTGGGCTTGTGTGGACCACTTTTGGTGGAGAGGTCCAGTTTGTGGAGGCTACAGCCATGGTAGGAAAGGGTGAACTTCATCTTACTGGGCAACTTGGTGACGTTATTAAAGAATCAGCACAAATAGCACTGACATGGGTAAGAGCCAGGGCAAAAGATCTCAAGTTGGCGGCTGCTGAGGAAATTAATCTGCTTCAGGGTCGGGATATTCACATACATTTTCCTGCTGGTGCTGTACCTAAGGATGGGCCCTCAGCAGGTGTGACTCTGGTCACAGCCTTGGTTTCACTGTTCAGTCAGAGAAGAGTAAGAGCAGATACAGCTATGACCGGAGAGATGACTTTGAGAGGTCTCATACTACCTGTTGGCGGTATCAAGGATAAGATTTTGGCAGCTCATCGATCTGGTATCAAAAGAGTTATCCTGCCACAGAGGAATTTGAAGGATTTAGTTGAAGTACCATCAACCGTGCGTTCCAGTCTTGAGATACTACTTGCCAAACGAATGGAAGATGTGTTGGAGCAGGCTTTTGAAGGGGGGTGCCCTTGGAGACAATACTCAAAATTATGACAGGACCCTCTGGGCATTGTAGCAATTTTTGATGATCTAAAAGCTTGTCACCTACAAATTTCATATTTCTCTGTAGCTGCTTTCTGTGGTCGGAGGTTAAAGAAATTATTGAGGAGTTCGTTTGACTGTTCATCGTCGAGCAATGAATGTTTAAGGACTTGGTGATCGATACACAGCCCTACTGAAGTTTCTGATGGAAATTCTTGGCATGTATTATTCCTTATTCCAATCAAGCTGTATCCATCTAGGGTAAGCAGCTGCTTCCCTCTTCTATTTTCTTCTATTTTATTTTATTTTATTTTTGTGGACAGGAATTACCTTTGAATGAGAGAATTGAGGCTCCCACGGAGGCGAAGAAGCCGAAGAAAGGAGAAGCCGGAGATGAGGATGTGGACATTGGCGATGATATGCCAATGAGCAATTTTCCACCTGTGGAGATTGAGAAGGATGATGGGCGGGCTGCTAGTGGCAGTTCTAGCAGTTCCAGCGGTTCAAGCAGTGGTTCTTCCTCATCTAGTGGTTCTGATTCTGGAAGTTCTTCAGGGAGTGATTCGGATGAGGATGATGCCAGGTCTTAGGAAGCATGACTTTTGGGGATTCATTTATTTTCTAAATACCCAAGTGGGTGGGTGCGTGTTTGTTTGAATGCTCGAGAGGAAGAAAGAGGGGGCCTTTATTAAGGTTGGGTTGAAATGAATGAGGTATAAATTACTCTAAACCTGTTTTCTATTTAATGCCTTTTCATTTCTTTAATGATGTTGGTGATAATTATGGTGAACGAATATAAACTCTTTCTTGTTGTGCAGAGTTGGGTTTTGAGGTGTGATTATGTCACTTAAGTGATTTGTGAAGAAGGACGGGAATTGGAAAGAGGGCACCATTGCTGGAGCATTGAAGTGTGAGAATATGTTTGAAGGACATGGATTCAGGCCTTCAAAAAAAAAAGGAAAAAAGGAAAGTGTAGAAGAAAGAGGGGAGATTATATTTGTACACATTACATAGACTAAATTAATACATGGGTTTTCTGATCAGAGTTCAATTCCTCTTACTTAAGAATGGCAGAAATTAAGATAGCTTTAAAATGTGAATGAACAGAACCAGAGCCCTTTTTGTCAACACCCAAAAAAATGGAAAGGAACGGGGGAATTTCGCGAGCATTCTTTCATAGGCGGATAAGAGATTGTTTGTAGTGACACTTAATTTATTGAGAAAGAGTTTGTTAGCCAGAGTTTTCATATAGATCCATTTTAATGATTCAATAAGCCAGTGTTGATTTTATAAAAGGTT

>*E.konishii* glutathione-S-transferase tau 1

CAACACTAACACGACCCATTTTTAATTCGTGTAAAAAATAATCAACACTAACACTAATTTTTTGTATTGTATTTGTATCGAATTGACGTGTTATGACTTATGTTAGCAGGCCTAACATTCATACTAAGGTAGAACATTTCCTTAGTTTCAAAAGGTTGCGCCTATGGTCAAGATCACTTCAGTTCTATTATTTTTATTCTCCTTAACTATCAACACGCACAACAACAGTGTATTTCTTTATTACAGAGAATATTTAGTCCACAAAGAAAACACAACTCAACTACAACAGTTCAAAATGCAACCAGATCTCAAAAGCTTAGTAGTATATGCTGGAGTTGGTTCTTCAGGCTGGACCATCCCTTTTCTTCAATACCAAAGCAAACTCATACACTTTGTTCTCATCAGCAAGAGAGTCAGAGACAGTCTTCCTGGTCATGCACCTCTTAGCCCATGCAATCAGCGCGGGACACTCTGCCTCTGTGCTGAAGTTACCCAAAGTCTCAAAGGCATAAAACCAGCAGTAGTATGGAATCAGAGCAACGTCCACAAACCCAAAGGTCTCTCCTCCAAAGTACGGCTTGTCTCCTAATTCCCCTTCCAACAATTTAAGGGCTTCTATTAATCCCTTTGTGCTTGCCTCATGCTCTTCTCCTTTTGTTGTCCATGTCTTCCTCCCGAGATCGTAGATCTTCTTGTCAATGAAGTCAGCCCAGAACCTAGCTTGAGCTCTGGGGTAAGGATCAGAGGGAAGCAATGGAGCTTTATCCTTCCAAACCTCATCAATATACTGCACAATGATTAGGGACTCAGAAATAGGTTTTCCATTGTGAATCAGAACTGGGACTTTTTTATGAATCGGATTCATCTCCAAAAGCAGATCGCTCTTCTTCCCCAACAAATCCTCCTCCCTATACTCATAATTCACTTCCTTCTCCGCCAGTGCTATTCTTGCCCTCATCCCAAACATACTCGCCCAGAAATCCAACAAAATAACCTCGTCCGCCATTGTTTTCCTGACAAATTTGCAGACAATGTTTTTGTGAGATTGTTTGCAGCGAATAGGAAGTGGAGATTTGTGAGTCAATTTATATAGTGGAAAAAAAGTGAGCTACGTATGGGAGAAAATTTTCTATGAACAATTACGCCACGTAGAATTTGCAACCGGCCAATAAAACTGTAGTATTTTAACCCTCAAACAATAATATGTCCCACAGGTGCACACTTGATCAGCTGCCTTGCTTTCCTTTTTGCACCTCCGTTTGCACACGCCTCACTATTGGTAGTAATTAGTCTCTGTTTTTTTGTTTTTATTTCAAATGTTAAATAAGTTAAGACACT

>*E.konishii* Cinnamyl alcohol dehydrogenase 1

TTCTTTTTCCCAATTACGGCCAAGTGCCTACCTATCAGCATGTCAATTTCATACAAAATTTCTTACAAAATTTGAAAGCATCCCACCAAAAAAATTGTGAACCACCAAACAAGTCAGTACAAATAGACGGGCAATAACAACAGATTTCTAGATCATGTAATAGCCAAAAAGTTTGGTATCTTTCAATCTCATCACCAACCAATTACCAACCCGTGTCCATATCTTCCGCACAAGTCACCATATCAAATTATAATATATGAATATATGAATTAGAACATTTCCATCAATCTTTGTCTTCATTTTTATTACCCAAAAGCTGAGAAAACAGAGCACTCATACTATTCCTATTTCACAACAAGAGTCCAAAAACAGAGCACTCAAAAACAAACACATAATTGAAGTTTAAATATGTTCTTGGCTATTTTTTTTTTTTTTGGGGATAATTTTCTTTACTGATCAAGATCAAGCTTGCTGCCGGCAACGTCTACGACGAACCTGTATCGAACATCATTCTTTTCGAGCCTTTCGAAAGCAGTGTTGATGTAATCCATTTTGATGACTTCAATCATGGAAGTGAGTCCCTTGTCTTTACAGAATTCTAGCATTTCCTCTGTTTCCTTCATGCTTCCAACAAAGCTCCCTGTGATGGCCTTTCTCCCAAGCATAACCATGGGACTAACAAACTGCAAAGGAGTATTAATAACGCCCATCAAGATCAACTTCCCATCAAGCTTCAACAAAGAAAGATACGGTTCAAGAGGATGGAATACCGGCACAGTGTCAATGATGTAGTCAAGTGAATCAGCAGCCTCTTGCATGCGAGTTTGGTCCGAGCTAACCAAATATTCATCGGCTCCGAGATGCTCCAATGCCTCCACTCTCTTCTTATCAGAAGAACTTATAACAGTCACATGGTGTCCCATTGCTTTGGCTATTTTCACCCCCATGTGTCCCACTCCTCCAAGCCCCAAAATTCCTCCTCTTAGACCACTTTGATTCAAGCCAAAGTGGGTCAGTGGACTGTACACTGTCACTCCGGCGCACAGCAGCGGTGCAGCCTGTTCCGGGGCCATTCCATCTGGGATTTTCACCACAAACCTTTGATCGACAACCATGGCGCTGGCAAAGCCGCCTTGGGTGGGCTTTCCGTCAGTGTAGACATCGTTGTACGACCATATCTTCTTGTTGCAGTATTGCTCAATGTCTGAATTGCAAGGACGGCAATTCCTGCAGCTTCCCACGATAGCTCCAACTCCGACCACATCTCCCACCCTGTATTTGGTTACCTCTGATCCCACGTCCGTCACCTCACCCACCACTTCATGCCCAGGAACCATGGGGTAGTGGGACATGCCGAGATCATTTTTGATCTGATGAATATCGGTATGGCAAATTCCACAGCATAGCACCTTCACGTAAACATCTTCTGGGCCTGTGCTTCTAAGACTGTAAGTATAAGGTGAAAGAATTCCAGATGGGTCTCTTGCTGCCCATCCAACTATTGTTTTCTCTTCTTCAAGGCTGCCCATTTCCTCCAACAAAGAAATATGGTTTGTTGGGGAAAAAGAAAAGCATACAGAGTGTATATATACACAGTGAGAGGTGGAGAGAATGAAGGCTGAAGACAAAAAGCAAAAGCAAAGTAGAAAGTAGTATGCGAAATCGAATATGTCTCGGGGTAGGGGCGACCCGCGCGGTA
