# Supplementary material for: Selection and evaluation of reference genes for qRT-PCR analysis in Euscaphis konishii Hayata based on transcriptome data
Source: Plant Methods. 2018 Jun 4;14:42. doi: 10.1186/s13007-018-0311-x (PMC5985561; doi:10.1186/s13007-018-0311-x)
Supplement: Supplementary file 2 — Additional file 2. Alignment and phylogenetic tree of 12 candidate genes and 1 validation gene. [file 13007_2018_311_MOESM2_ESM.docx]

**Figure S1 Sequence aligement of *E. konishii* Ubiquitin-conjugating enzyme E2-17 kDa (*EkUB*C)**


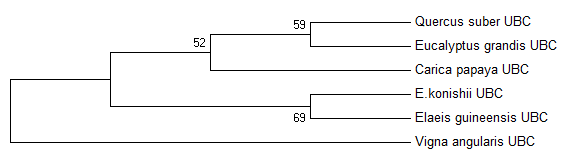


**Figure S2 Phylogenetic tree of *E. konishii* Ubiquitin-conjugating enzyme E2-17 kDa (*EkUBC*) (**The evolutionary history was inferred using the Neighbor-Joining method. The bootstrap consensus tree inferred from 1000 replicates is taken to represent the evolutionary history of the taxa analyzed. Branches corresponding to partitions reproduced in less than 50% bootstrap replicates are collapsed. The percentage of replicate trees in which the associated taxa clustered together in the bootstrap test (1000 replicates) are shown next to the branches. The evolutionary distances were computed using the Maximum Composite Likelihood method and are in the units of the number of base substitutions per site. Evolutionary analyses were conducted in MEGA7. And the same was below.**)**

**Figure S3 Sequence aligement of *E. konishii* F-actin capping protein alpha subunit (*EkF-ACP*)**

**
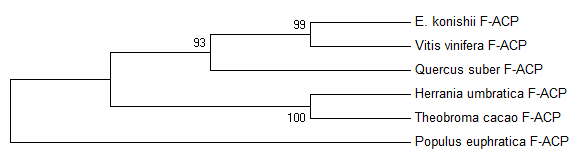
**

**Figure S4 Phylogenetic tree of *E. konishii* F-actin capping protein alpha subunit (*EkF-ACP*)**

**Figure S5 Sequence aligement of *E. konishii* Actin-related protein 7 (*EkARP7*)**

**
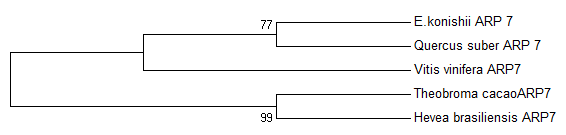
**

**Figure S6 Phylogenetic tree of *E. konishii* Actin-related protein 7 (*EkARP7*)**

**Figure S7 Sequence aligement of *E. konishii* Elongation factor 2 (*EkEF2*)**

**
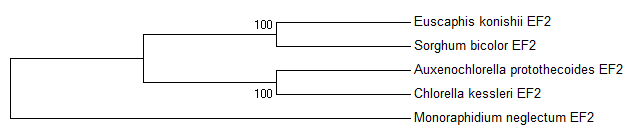
**

**Figure S8 Phylogenetic tree of *E. konishii* Elongation factor 2 (*EkEF2*)**

**Figure S9 Sequence aligement of *E. konishii* Actin (*EkACT*)**

**
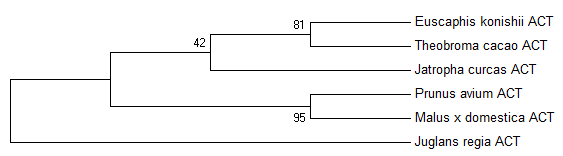
**

**Figure S10 Phylogenetic tree of *E. konishii* Actin (*Ek*ACT)**

**Figure S11 Sequence aligement *E. konishii* Glyceraldehyde-3-phosphate dehydrogenase (*Ek*GADPH)**

**
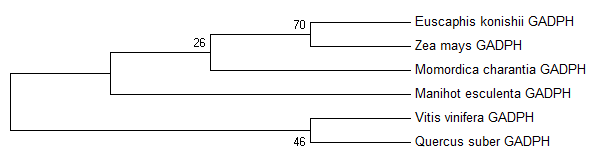
**

**Figure S12 Phylogenetic tree of *E. konishii* Glyceraldehyde-3-phosphate dehydrogenase (*Ek*GADPH)**

**Figure S13 Sequence aligement of *E. konishii* Eukaryotic elongation factor 5A-1 (*EkEEF-5A-1*)**

**
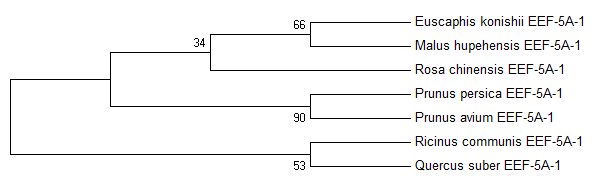
**

**Figure S14 Phylogenetic tree of *E. konishii* Eukaryotic elongation factor 5A-1 (*EkEEF-5A-1*)**

**Figure S15 Sequence aligement of *E. konishii* Actin-depolymerizing factor 2 (*EkADF2*)**

**
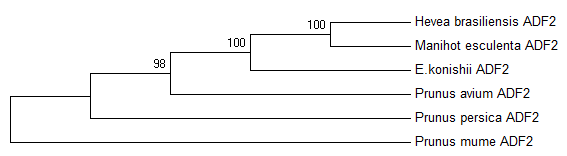
**

**Figure S16 Phylogenetic tree of *E. konishii* Actin-depolymerizing factor 2 (*EkADF2*)**

**Figure S17 Sequence aligement of *E. konishii* *β*-Tubulin (*EkTUB*)**

**
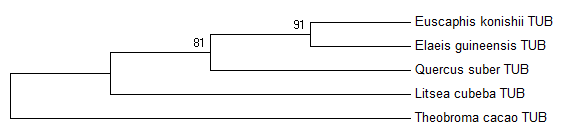
**

**Figure S18 Phylogenetic tree of *E. konishii* *β*-Tubulin (*EkTUB*)**

**Figure S19 Sequence aligement of *E. konishii* PLAC8 family protein isoform 2 (*EkPLAC8*)**

**
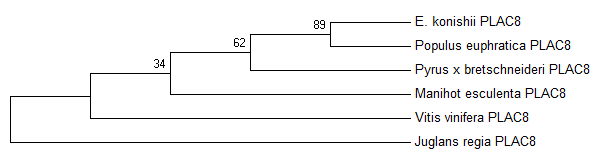
**

**Figure S20 Phylogenetic tree of *E. konishii* PLAC8 family protein isoform 2 (*EkPLAC8*)**

**Figure S21 Sequence aligement of *E. konishii* Lonprotease-2-like protein (*EkLPP*)**

**
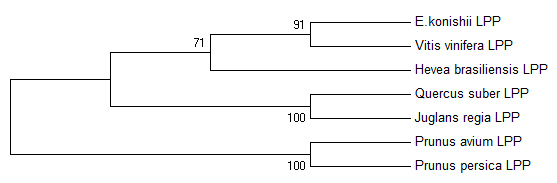
**

**Figure S22 Phylogenetic tree of *E. konishii* Lonprotease-2-like protein (*EkLPP*)**

**Figure S23 Sequence aligement of *E. konishii* Glutathione-S-transferase tau 1 (*EkGSTU1*)**

**
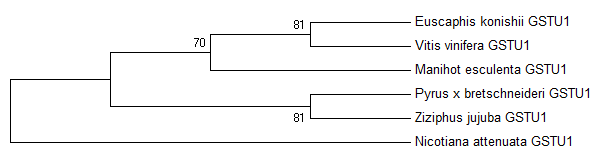
**

**Figure S24 Phylogenetic tree of *E. konishii* Glutathione-S-transferase tau 1 (Ek*GSTU1*)**

**Figure S25 Sequence aligement of *E. konishii* Cinamyl alcohol dehydrogenase 1 (*EkCAD1*)**

**
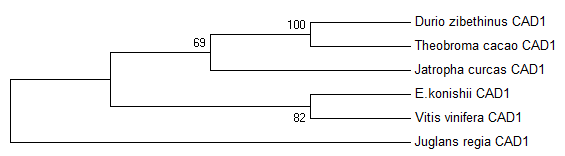
**

**Figure S26 Phylogenetic tree of *E. konishii* Cinamyl alcohol dehydrogenase 1 (*EkCAD1*)**
