# Supplementary figures and images for: Selection and evaluation of reference genes for qRT-PCR analysis in Euscaphis konishii Hayata based on transcriptome data
Source: Plant Methods. 2018 Jun 4;14:42. doi: 10.1186/s13007-018-0311-x (PMC5985561; doi:10.1186/s13007-018-0311-x)

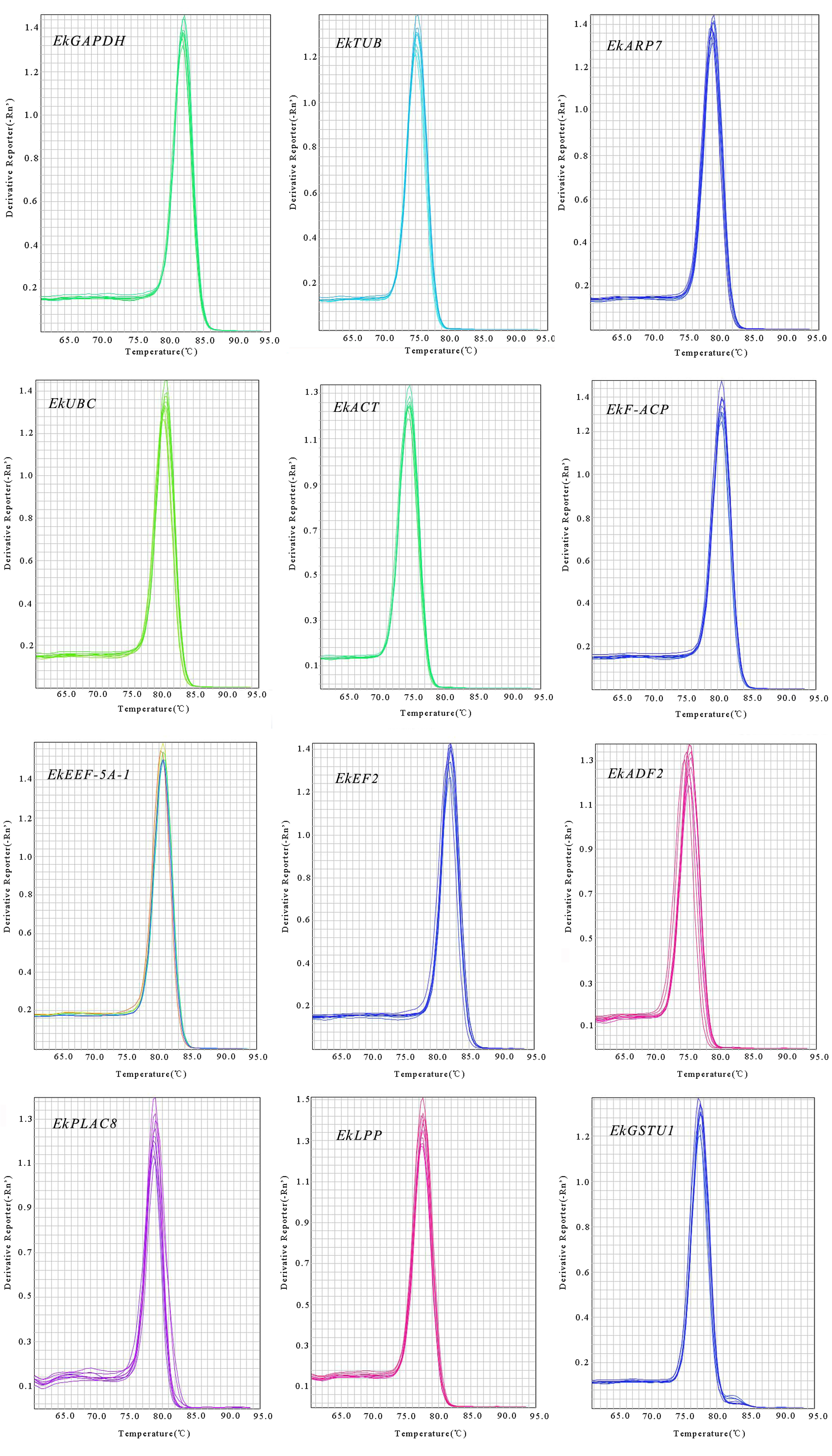

Supplement: Supplementary file 3 — Additional file 3. Melting curves for the 12 candidate reference genes. [file 13007_2018_311_MOESM3_ESM.jpg]
